# Skin cancers and their risk factors in older persons: a population-based study

**DOI:** 10.1186/s12877-022-02964-1

**Published:** 2022-04-01

**Authors:** Suvi-Päivikki Sinikumpu, Jari Jokelainen, Sirkka Keinänen-Kiukaanniemi, Laura Huilaja

**Affiliations:** 1 Department of Dermatology, Oulu University Hospital, P.B.20, FIN-90029 OYS, Oulu, Finland; 2 Medical Research Center, PEDEGO Research Group, University of Oulu, Oulu, Finland; 3 Northern Finland Birth Cohorts, Arctic Biobank, Infrastructure for Population Studies, Faculty of Medicine, University of Oulu, Oulu, Finland; 4 Center for Life Course Health Research, Faculty of Medicine University of Oulu, Oulu, Finland; 5 Healthcare and Social Services of Selänne, Pyhäjärvi, Finland

**Keywords:** Skin cancer, Total body skin examination, Screening, Risk factors

## Abstract

**Background:**

The number of skin cancer is increasing rapidly. However, little is known about the risk factors of skin cancer in older persons. Our objectives were to determine the risk factors for skin cancer or its precursors in an older population. More specifically, to study the association of new skin cancers with previous skin cancer, sex, age, Fitzpatrick’s skin type, history of outdoor work and socioeconomic status (SES).

**Methods:**

In this retrospective cross-sectional study of a large, well documented historical cohort data set a total body skin examination (TBSE) was performed for 552 participants aged between 70 and 93 years by dermatologists. The information gathered was augmented with health register data and self-reported data. The associations between skin cancer and its risk factors were studied by using the logistic regression analyses.

**Results:**

According to the TBSE skin cancer/precursor was present in 25.5% of participants and was more common in males than in females (34.5% vs 20.2%, *p* < 0.001). Previous skin cancer increased the risk of subsequent skin cancer 2.6-fold (OR 2.56, 95% CI 1.43-4.55) and male sex nearly 2-fold (1.97, 95% CI 1.26-3.08). Specific risk factors for the first occurrence of skin cancer were male sex and outdoor work. There was also association between skin cancer and age and socioeconomic status.

**Conclusions:**

TBSE is recommend for physicians treating older persons to allow early recognition of skin cancers or their precursors. Older males need particularly close attention.

**Supplementary Information:**

The online version contains supplementary material available at 10.1186/s12877-022-02964-1.

## Introduction

The incidence of melanoma and non-melanoma skin cancers is rising annually [1]. Today, skin cancers are the most common diagnosed malignancy in developed countries [2]. Age is indisputable a major risk factor of skin cancer [3], and, since the life expectancy has risen [4], the number of skin cancers is expected to increase unless more effort is made towards their early recognition and prevention.

Despite the increasing incidence of skin cancer, it remains unclear whether skin cancer screening – performed by total body skin examination (TBSE) – is effective or not [5, 6]. The Northern Germany skin cancer project concluded that skin cancer screening could enhance the early recognition and treatment of skin cancers [6]. On the other hand, the US Preventive Services Task Force (USPSTF) deduced that there is insufficient evidence that benefits of TBSE to screen for skin cancer outweigh the risks [7]. However, also USPSTF agrees that targeted risk groups could benefit from skin cancer screening [5]. In a Finnish study (*n* = 4037, mean age 69 years) a high proportion (38%) of participants who were referred to a Department of Dermatology for skin malignancy presented with a new additional skin cancer when screened by dermatologists. In that study malignant tumors were found most commonly in older men [8].

The present study was designed to evaluate possible risk factors for skin cancer in a general population of older persons by using the unique data of the Northern Finland Birth Cohort Parents’ Study and the Finnish Care Register for Health Care (CRHC). The associations of new skin cancer with previous skin cancer, sex, age, Fitzpatrick’s skin type, the history of outdoor work and socioeconomic status (SES) were studied.

## Method

### Setting and participants

The Northern Finland Birth Cohort 1966 (NFBC1966) is an epidemiological and longitudinal research program in the two northernmost provinces in Finland (Oulu and Lapland). The original cohort was created by identifying all pregnant women living in the specified geographic area whose expected delivery date fell between 1st January and 31st December 1966. Altogether, 12,055 mothers with 12,058 live born children formed the study population (covered 48% of Finnish territory in 1966). The children of NFBC1966 have been followed regularly since their birth and their mothers have been followed since, on average, the 16th week of pregnancy [9, 10].

In 2018, a comprehensive health questionnaire was sent to all the parents of the NFBC1966 cohort members, who were known to be alive and still living in the Oulu area. All those who returned the questionnaire were invited to a clinical examination. The subset of parents who participated in the clinical examination is referred to as the ‘Parents study’ cohort (Keränen et al., unpublished observations). The data were collected between May 2018 and March 2019 on the premises of the Faculty of Medicine of the University of Oulu [11].

### TBSE

**Fig. 1:**
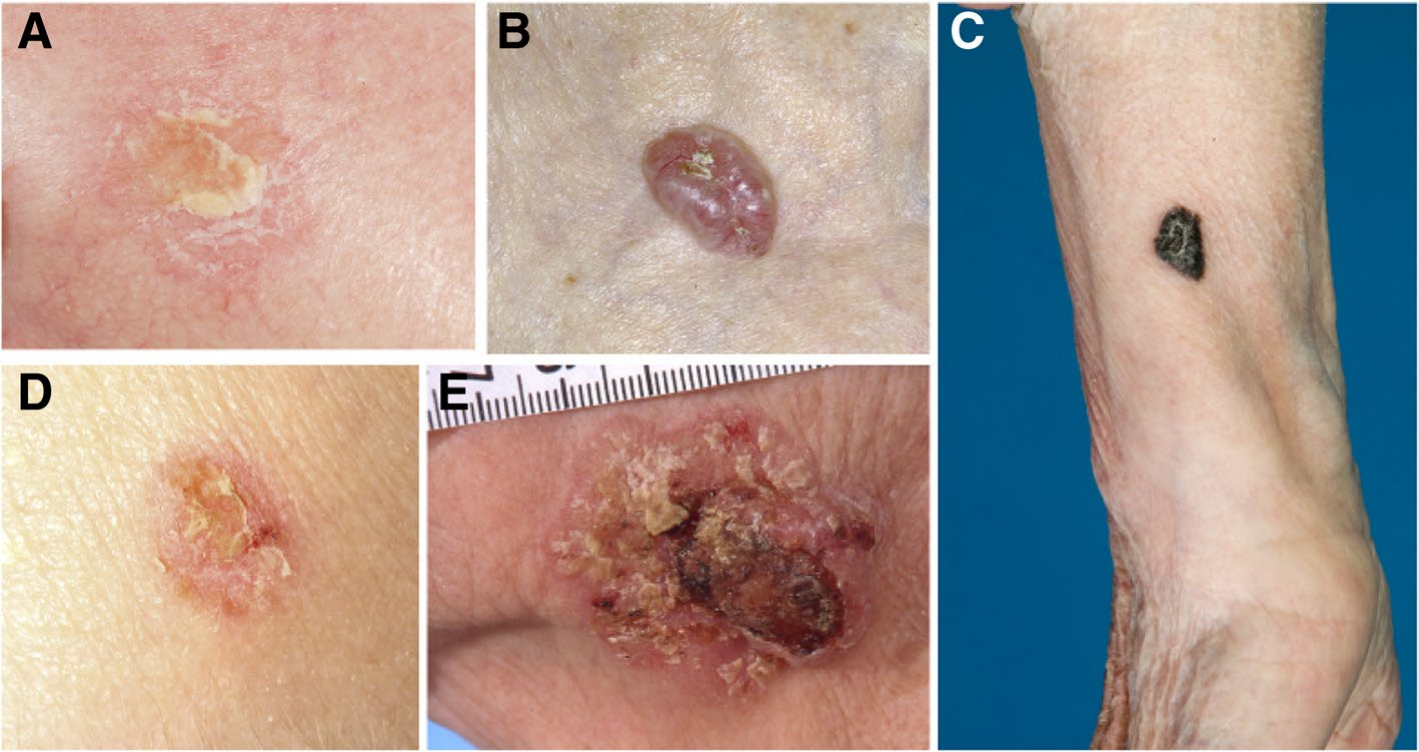
Clinical presentations of skin cancers or their precursors. **A** Actinic keratosis; **B** Basal cell carcinoma; **C** Melanoma; **D** Bowen’s disease; **E** Squamous cell carcinoma. Photos: Archives of the Department of Dermatology, Oulu University Hospital

As part of the clinical study a dermatological whole skin examination was performed by dermatologists (SPS, LH). All skin areas were observed including the nails, hair and scalp. Skin tumors (Fig. 1) were counted and further observed using a dermatoscope. The dermatological evaluation was performed carefully, with 20 min time booked for each case. Diagnoses were based on internationally accepted criteria and classified by the International Classification of Diseases (ICD) [11].

In order to analyze the severity of skin diseases or skin findings, immediately after the TBSE the study participants were further classified into three subgroups according to their need of further interventions: I) no further care needed, II) expected to recover with self-treatment, III) need medical care by physician. If a study case showed any skin disease that required treatment (e.g. untreated eczema) or had any suspected skin malignancy, the participants was referred to a primary health care unit. Individuals with only benign skin tumors and/or male/female pattern hair loss were categorized as “no further care needed” [11].

### History of previous skin cancer

Patient records (history of previous skin cancer) were obtained from the Finnish Institute of Health and Welfare’s statutory Care Register of Health Care (CRHC), and were selected by all International Classification of Diseases (ICD-9) codes for skin cancer (Please see, Supplementary data). The ICD-10 codes L57 actinic keratosis, C43-C44 basal cell carcinoma, squamous cell carcinoma, malignant melanoma, D03-D04 melanoma in situ and carcinoma in situ were also included. The CRHC contains inpatient data from all state-administered Finnish hospitals and from the largest private hospitals from 1987 onwards. Each record contains the identification numbers of the patient and hospital, primary and subsidiary diagnoses, and duration of hospital stay. The Care Register also covers outpatient visits from 1998 onwards and outpatient visits in primary health care since 2011. In Finland, ICD-9 was used between 1987 and 1995 and was replaced with ICD-10 in 1996.

As part of the clinical skin examination participants were asked whether they had ever received a diagnosis of skin cancer or a precursor, including actinic keratosis, basal cell carcinoma, squamous cell carcinoma, melanoma, melanoma in situ or carcinoma in situ (Self-reported data).

### Fiztpatrick’s skin type, history of outdoor working, SES and living status

Skin type was evaluated by both the TBSE findings and by self-reported data (participant’s own opinion of skin type). The study participants were classified into four groups according to their skin type by using the modified Fitzpatrick’s criteria as follows: skin type I “skin always burns”, type II “skin burns often”, type III “skin burns occasionally” and type IV “skin never burns” [12].

The information on outdoor working was gathered by asking the participants the following question: “Has your principal work been outdoors?” (Yes or No). The study participants were classified into three subgroups based on their SES, defined by their highest level of education [13]: primary school; secondary school; post-secondary level education/vocational college/university. The information on outdoor work, education and living status (living alone or with a spouse/other family member) of study participants were self-reported.

### Definitions

In a further analysis, the term *‘Lifelong skin cancer’* included any diagnosed skin cancer or its precursor that was found by the TBSE or in the CRHC or was self-reported. ‘*First skin cancer ever’* in this study means that the person received their first skin cancer or precursor diagnosis during the study TBSE, in the absence of any history of skin cancer.

### Confounding factors

Age, outdoor work and skin type were considered to be confounding factors because they are known risk factors of skin cancers [14–16].

### Statistical analyses

The overall prevalence of skin cancers and their precursors was calculated. The Chi-Square test was used to test differences in categorical variables. Logistic regression analyses were used to estimate crude and adjusted odds ratios (OR) and their 95% confidence intervals (CI). The following variables were used in the adjusted multivariate model: sex, age, outdoor working, Fitzpatrick’s skin type, socioeconomic status, previous history of skin cancer (CRCH and self-reported data). The statistical analyses were conducted using the R software package version 4.0.2 (https://cran.rstudio.com). All significance tests were two-tailed, and values of *p* ≤ 0.05 were considered statistically significant.

### Ethical approval

The Ethical Committee of the Northern Ostrobothnia Hospital District approved the study (115/2012) which was performed according to the principles of the 1983 Declaration of Helsinki. Informed written consent to participate was obtained from all study participants. The data were handled on a group level only, personal information being replaced by identification codes resulting in complete anonymity.

## Results

### Characteristics of the study population

A total of 12,027 parents of NFBC1966 were sent a diverse health questionnaire. Of them 5158 answered (43%). All those who were living in Oulu area and answered to the health questionnaire were invited to participate to the clinical examination (*n* = 1256). In total, 684 agreed. The final analysis population consisted of the 552 participants (*n* = 346 females and *n* = 206 men) who received the TBSE. Participant age ranged between 70 and 93 years; 19.6% (*n* = 108) were aged 70–75 years, 42.4% (*n* = 234) 75–80 years, 29.3% (*n* = 162) 80–85 years, 7.8% (*n* = 43) 85–90 years and 0.9% (*n* = 5) over 90 years. The baseline characteristics of the study members are presented in Supplementary Table 1.

### Prevalence of skin cancers or their precursors

**Fig. 2:**
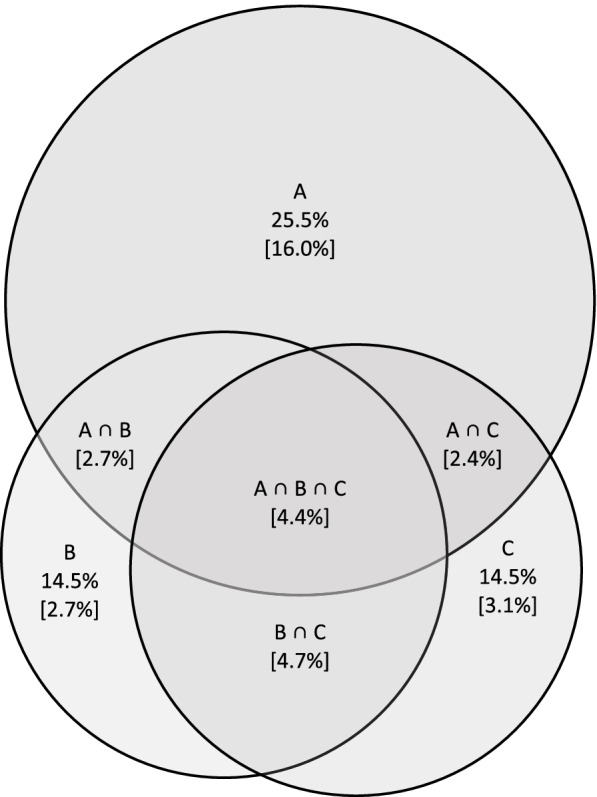
Prevalence of skin cancers or their precursors in clinical skin examinations, in self-reported data and in registries. In parenthesis: pure or combined prevalence. **A** Skin cancer on clinical examination; **B** Self-reported skin cancer; **C** Skin cancer on CRHC

Detailed numbers of skin cancers and precursors found by the TBSE and in the CRCH are shown in Table 1. According to the TBSE the prevalence of skin cancer or its precursors was 25.5% (*n* = 141/552) while there were 165 skin cancers or precursors in total. The TBSE revealed *first skin cancer ever* in 88 participants (Supplementary Table 2). There were 80 participants who self-reported historical skin cancer (data not shown). Overlapping of skin cancers or their precursors are shown in Fig. 2.

**Table 1:** The number of participants with skin cancers or precursors

	Total body skin examination	Care Register of Health Care data
	*n* = 141	*n* = 80
Actinic keratosis	123 (87.2%)	37 (46.2%)
Bowen’s disease	9 (6.38%)	2 (2.50%)
Melanoma	3 (2.13%)	1 (1.2%)
Basal cell carcinoma	28 (19.9%)	29 (36.2%)
Squamous cell carcinoma	2 (1.42%)	6 (7.5%)
Melanoma in situ		1 (1.25%)
Unspecified skin cancer^a^		6 (7.5%)

^a^Unspecified due to missing histological classification

### Risk factors for skin cancer or their precursors based on TBSE findings

According to the TBSE findings, skin cancers or their precursors were more common in males (34.5%) than females (20.2%; *p* < 0.001). Those study participants diagnosed with skin cancer during the TBSE had significantly more skin cancers in their history (46.2%) than those whose TBSE found no skin cancers (22.0%) (*p* < 0.001). Logistic regression analyses showed that previous skin cancer increased the risk of subsequent skin cancer 2.6-fold (OR 2.56, 95% CI 1.43-4.55) and male sex nearly 2-fold (OR 1.97, 1.26-3.08; Table 2).

**Table 2:** Risk factors of skin cancer or their precursor by total body skin examination findings

	Skin cancer/precursor in TBSE		OR (univariable)	OR (multivariable)^b^
	No n(%)	Yes n(%)		
Sex				
Female	276 (79.8)	70 (20.2)	Ref	Ref
Male	135 (65.5)	71 (34.5)	2.07 (1.41-3.06, *p* < 0.001)	1.97 (1.26-3.08, *p* = 0.003)
Age				
Mean (SD)	75.7 (4.2)	76.9 (4.2)	1.06 (1.02-1.11, *p* = 0.007)	1.04 (0.99-1.10, *p* = 0.123)
Outdoor working				
No	360 (75.9)	114 (24.1)	Ref	Ref
Yes	51 (66.2)	26 (33.8)	1.61 (0.95-2.68, *p* = 0.071)	1.71 (0.93-3.10, *p* = 0.079)
Fitzpatrick’s skin type				
I-III	323 (73.4)	117 (26.6)	Ref	Ref
IV	86 (78.2)	24 (21.8)	0.77 (0.46-1.25, *p* = 0.306)	0.71 (0.39-1.24, *p* = 0.239)
Socioeconomic status				
No education/Primary school	85 (81.7)	19 (18.3)	Ref	Ref
Secondary school	149 (77.2)	44 (22.8)	1.32 (0.73-2.45, *p* = 0.363)	1.21 (0.65-2.29, *p* = 0.558)
Post-secondary level education/vocational college/university	132 (69.5)	58 (30.5)	1.97 (1.11-3.60, *p* = 0.024)	1.79 (0.97-3.40, *p* = 0.066)
Previous skin cancer^a^				
No	368 (78.0)	104 (22.0)	Ref	Ref
Yes	43 (53.8)	37 (46.2)	3.04 (1.86-4.97, *p* < 0.001)	2.56 (1.43-4.55, *p* = 0.001)

There may be some missing data while not all participants reported complete information on health questionnaires

*OR* Odds ratio, *TBSE* Total body skin examination, *CRCH* the Finnish Care Register for Health Care)

^a^ Data from CRCH

^b^ Logistig regression analysis, adjusted for sex, age, outdoor working, Fitzpatrick’s skin type, socioeconomic status, previous history of skin cancer (the CRCH and self-reported data). A Wald z-statistic was used for *p*-values

### Risk factors of ‘lifelong skin cancer’

To analyze risk factors of *‘lifelong skin cancer’*, the findings of the TBSE were pooled with the data from registries and self-reporting. In this analysis higher age and SES were associated with skin cancers (*p* < 0.001, *p* < 0.05, respectively). Skin cancers were more frequent in participants with a history of outdoor working than in those who worked indoors (*p* < 0.05). In logistic regression analyses, age increased the risk of skin cancer 1.05-fold (OR 1.05, 95% CI 1.01-1.11), outdoor working nearly 2-fold (OR 1.92, 95% CI 1.10-3.37) and higher SES 1.70-fold (OR 1.70, 95% CI 1.00-2.95). Male sex increased the risk of skin cancer 1.5-fold but the finding was not statistically significant in the multivariate model (OR 1.49, 95% CI 0.99-2.23). There was no association between Fitzpatrick’s skin type and skin cancers. Table 3.

**Table 3:** Risk factors for ‘lifelong skin cancer’

	Lifelong skin cancer or precursor^a^		OR (univariable)	OR (multivariable)^c^
	No *n* (%)	Yes*n* (%)^b^		
Sex				
Female	236 (68.2)	110 (31.8)	Ref	Ref
Male	117 (56.8)	89 (43.2)	1.63 (1.14-2.33, *p* = 0.007)	1.49 (0.99-2.23, *p* = 0.053)
Age				
Mean (SD)	75.6 (4.1)	76.8 (4.3)	1.07 (1.03-1.12, *p* = 0.001)	1.05 (1.01-1.11, *p* = 0.027)
Outdoor working				
No	312 (65.8)	162 (34.2)	Ref	Ref
Yes	41 (53.2)	36 (46.8)	1.69 (1.04-2.75, *p* = 0.034)	1.92 (1.10-3.37, *p* = 0.022)
Fitzpatrick’s skin type				
I-III	277 (63.0)	163 (37.0)	Ref	Ref
IV	74 (67.3)	36 (32.7)	0.83 (0.53-1.28, *p* = 0.400)	0.92 (0.56-1.49, *p* = 0.736)
Socioeconomic status				
No education/Primary school	74 (71.2)	30 (28.8)	Ref	Ref
Secondary school	130 (67.4)	63 (32.6)	1.20 (0.71-2.03, *p* = 0.501)	1.19 (0.70-2.05, *p* = 0.528)
Post-secondary level education/vocational college/university	111 (58.4)	79 (41.6)	1.76 (1.06-2.96, *p* = 0.032)	1.70 (1.00-2.95, *p* = 0.055)

There may be some missing data while not all participants reported complete information on health questionnaires

*CRHC* the Finnish Care Register for Health Care, *SD* standard deviation, *TBSE* total body skin examination, *OR* odds ratio

^a^Data from CRHC, TBSE and from self-reporting

^b^There can be some missing data while all participants have not given a permission to use CRHC data

^c^ Logistic regression analysis, adjusted for sex, age, outdoor working, Fitzpatrick’s skin type, socioeconomic status, previous history of skin cancer (CRCH and self-reported data). A Wald z-statistic was used for *p*-values

### Risk factors of subpopulation with ‘first skin cancer ever’

Finally, in a separate analysis of the subgroup of participants with ‘*first skin cancer ever’* found by the TBSE, males had more first skin cancers than females (27.8% vs 15.4%, OR 1.85, 95% CI 1.09- 3.15). A history of outdoor working also increased the risk of first skin cancer (OR 2.31, 95% CI 1.16- 4.52; Supplementary Table 2).

## Discussion

This population-based study of 552 participants aged over 70 years found a high prevalence of skin cancers or their precursors (25.5%). We demonstrated that a history of previous skin cancer increased the risk of new skin cancer 2.6-fold in this population. Other risk factors of skin cancers or their precursors were male sex, older age, higher SES and a history of outdoor working. The specific risk factors among those with *first skin cancer ever* were male sex and outdoor working.

Advanced age is a significant risk factor for all skin cancers [17]. Non-melanoma skin cancer (NMSC; including squamous cell carcinoma [SCC] and basal cell carcinoma [BCC]) is the most commonly diagnosed cancer worldwide and particularly affects older persons [3, 18]. The incidence of melanoma and its associated mortality rate also increase with age and new diagnoses are most typical in those aged over 75 years [15]. In line with previous studies, we found that older age was associated with a greater risk of skin cancer. Even though best practices are yet to be determined for skin cancer screening, many studies have highlighted the importance of TBSE in older persons [3, 8].

In our study a history of skin cancer increased the risk for new skin cancers, a finding that also corresponds with previous reports [8, 19–21]. According to meta-analyses by Stern and Marcil previous skin cancer is often followed by subsequent skin cancers, particularly in cases of BCC and SCC [19]. In a Finnish study an additional new skin cancer was found from 38% of the patients who were referred to the Department of Dermatology because of skin cancer [8]. In a retrospective study from Portugal (*n* = 969) 17% of patients with previous skin cancer developed a new consecutive skin malignancy and this risk particularly increased with advanced age [20].

We want to emphasize that in our study there were as many as 88 study cases who were diagnosed with ‘*their first skin cancer ever’* during our TBSE but who were totally unaware of the presence of skin malignancy. The majority of these participants were males, possibly reflecting the fact that men are generally less likely than women to seek help from physician [22]. It is noteworthy that male sex is associated with a higher risk for all types of skin cancer when compared with female sex [8, 23]. Older men are also the group at highest risk for death from melanoma [23, 24].

The US Preventive Services Task Force (USPSTF) found insufficient evidence to support the regular use of the TBSE [7]. However, it agreed that targeted research among populations with the highest skin cancer burden would be useful [5]. Many cancers (including skin cancers) could be prevented or treated successfully if detected earlier. A TBSE can identify precursors of skin cancers, and treating precursors can often yield benefits years later. Furthermore, the costs of skin cancer are highest among patients diagnosed with late-onset disease (11), and diagnosis at a later stage of disease correlates with a poorer survival rate (12). Based on the findings of the present study and previous literature, older men carry the highest risk of skin cancer which should keep in mind [8].

In line with previous results [14] we also found that a history of outdoor working and thus, high cumulative exposure to ultraviolet radiation, increased the risk for skin cancer in older participants. However, we did not find any association between Fitzpatrick’s skin type and skin cancers, even though it is well known that persons with a fair complexion have an increased risk for skin cancer [16]. This may originate from the fact that we had too little variability to detect differences in skin types.

The primary strength of the present study is its unselected population of older persons belonging to the NFBC Parents’ study. In comparison, previous studies have focused on narrower, more selected populations, such as patients at dermatologic clinics or tertiary care centers – approaches that may have been prone to selection bias [24, 25]. The other major strength is that the TBSE was performed on all participants by experienced dermatologists who have the best ability to screen skin findings. The clinical data were augmented by self-reported data and information from the CRHC, which has been shown to be a reliable and accurate source of diagnosis data [26]. Interobserver reliability was tested between the two main researchers (SPS, LH) and its degree was high [27]. Although all participants of the Parents’ study were invited to participate in the present analysis, not all did, which must be acknowledged as a potential study weakness as this may have caused sample selection bias. For example, most of the participants were still living in their own homes rather than nursing homes, which may represent a possible skew in the population. Furthermore, as seen in the main NFBC cohort, participants were more likely than non-respondents to be from a higher social class, and more likely to be living with a spouse/family members, rather than alone [28].

## Conclusions

As a conclusion, we found that many older participants were diagnosed with the first or subsequent skin cancer during the TBSE. These findings support the importance of skin evaluation of those with advanced age. More power should be focused on prevention of skin cancers. To answer this gap, European Skin Cancer Foundation has already launched prevention strategies like SunPass Kindergarten [29], and e.g.in Finland North-Savo Skin Cancer Program (NOSCAP) has focused both in primary (to find those in highest risk of skin cancer) and secondary prevention of skin cancers [30]. Physicians treating older persons are encouraged to perform a skin examination to detect skin cancers and their precursors. Previous history of skin cancer - which is easy to elicit from the patient - can act as a clue to the patient being at higher risk of new skin cancers. Because we found that skin cancer and precursors particularly affected men, we especially call for caution when treating older men. However, what comes to treatment of skin tumors of older person a patient’s personal situation must always be taken into consideration when planning treatment options. For frail patients with a limited life-expectancy, watchful-waiting can sometimes be a right option [31].

## Supplementary Information

**Additional file 1: Supplementary data. Supplementary Table 1.** Participants demographics and skin disease status. **Supplementary Table 2.** Risk factors of *‘first skin cancer ever’* diagnosed by TBSE*.*

## Data Availability

The data that support the findings of this study are available from Northern Finland Birth Cohort 1966 Study. Restrictions apply to the availability of these data, which were used under license for this study. The use of personal data is based on cohort participant’s written informed consent at his/her latest follow-up study, which may cause limitations to its use. Permission to use the data can be applied for research purposes via electronic material request portal. Please, contact NFBC project center (NFBCprojectcenter@oulu.fi) and visit the cohort website (www.oulu.fi/nfbc) for more information.

## Abbreviations

NFBC: Northern Finland Birth Cohort
TBSE: Total body skin examination
CRCH: The Finnish Care Register for Health Care
SES: Socioeconomic status
OR: Odds ratio
NMSC: Non melanoma skin cancer
