# Supplementary material for: Skin cancers and their risk factors in older persons: a population-based study
Source: BMC Geriatr. 2022 Apr 1;22:269. doi: 10.1186/s12877-022-02964-1 (PMC8973875; doi:10.1186/s12877-022-02964-1)
Supplement: Supplementary file 1 — Additional file 1: Supplementary data. Supplementary Table 1. Participants demographics and skin disease status. Supplementary Table 2. Risk factors of ‘first skin cancer ever’ diagnosed by TBSE. [file 12877_2022_2964_MOESM1_ESM.docx]

**Clear supplementary data**

**Skin cancers and their risk factors in older persons: A population-based study**

**Supplementary data**

Patient records (history of previous skin cancer) were obtained from the Finnish Institute of Health and Welfare’s statutory Care Register of Health Care (CRHC), and were selected by all International Classification of Diseases (ICD-9) codes for skin cancer (The ICD-10 codes L57 actinic keratosis, C43-C44 basal cell carcinoma, squamous cell carcinoma, malignant melanoma, D03-D04 melanoma in situ and carcinoma in situ were also included):

| ICD-10 | ICD-9 |
| --- | --- |
| L57 | 702.0 |
| C43 | 1720[A-D]  1721[A-D]  1722[A-D]  1723[A-D]  1724[A-D]  1725[A-D]  1726[A-D]  1727[A-D]  1728[A-D]  1729[A-D] |
| C44 | 1730B  1731B  1732B  1733B  1734B  1735B  1736B  1737B  1738B  1739B |
| C44 | 1730A  1731A  1732A  1733A  1734A  1735A  1736A  1737A  1738A |
| C44 | 1730C  1731C  1732C  1733C  1734C  1735C  1736C  1737C  1738C  1739C |
| D03 | 2320A  2321A  2322A  2323A  2324A  2325A  2326A  2327A  2328X  2329X |
| DO4 | 2320A  2321A  2322A  2323A  2324A  2325A  2326A  2327A  2328X  2329X |
|  |  |

**Supplementary Table 1.** Participants demographics and skin disease status

| Sex (N=552) | n (%) |
| --- | --- |
| Male | 206 (37.3%) |
| Female | 346 (62.7%) |
| Living status (N=549)^a^: |  |
| Living alone | 164 (29.9%) |
| Living with spouse or with other family member | 385 (70.1%) |
| Education (N=487)* |  |
| No education/Primary school | 104 (21.4%) |
| Secondary school | 193 (39.6%) |
| Post-secondary level education/vocational college/university | 190 (39.0%) |
| Age, years (N=552) |  |
| Mean (SD) | 78.4 (4.18) |
| Age range: |  |
| 70 to 75 | 108 (19.6%) |
| 75 to 80 | 234 (42.4%) |
| 80 to 85 | 162 (29.3%) |
| 85 to 90 | 43 (7.79%) |
| 90 to 93 | 5 (0.91%) |

SD, standard deviation

^a^Not all participants reported complete information on health questionnaires

**Supplementary Table 2.** Risk factors of *‘first skin cancer ever’* diagnosed by TBSE

|  |  | First skin cancer ever in TBSE | | OR (univariable) | OR (multivariable)^a^ |
| --- | --- | --- | --- | --- | --- |
|  |  | No n(%) | Yes n(%) |  |  |
| Sex | Female | 236 (84.6) | 43 (15.4) | Ref | Ref |
|  | Male | 117 (72.2) | 45 (27.8) | 2.11 (1.32-3.40, p=0.002) | 1.85 (1.09-3.15, p=0.022) |
| Age | Mean (SD) | 75.6 (4.1) | 76.8 (4.5) | 1.07 (1.01-1.13, p=0.019) | 1.06 (0.99-1.12, p=0.083) |
| Outdoor working | No | 312 (82.3) | 67 (17.7) | Ref | Ref |
|  | Yes | 41 (66.1) | 21 (33.9) | 2.39 (1.31-4.26, p=0.004) | 2.31 (1.16-4.52, p=0.015) |
| Fitzpatrick’ skin type | I-III | 277 (78.7) | 75 (21.3) | Ref | Ref |
|  | IV | 74 (85.1) | 13 (14.9) | 0.65 (0.33-1.20, p=0.187) | 0.69 (0.33-1.34, p=0.292) |
| Socioeconomic status | No education/Primary school | 74 (82.2) | 16 (17.8) | Ref | Ref |
|  | Secondary school | 130 (82.3) | 28 (17.7) | 1.00 (0.51-2.00, p=0.991) | 0.96 (0.48-1.97, p=0.904) |
|  | Post-secondary level education/vocational/  college/university | 111 (74.5) | 38 (25.5) | 1.58 (0.84-3.11, p=0.168) | 1.45 (0.73-2.99, p=0.303) |

TBSE, total body skin examination

^a^  Logistic regression analysis, adjusted for sex, age, outdoor working, Fitzpatrick’s skin type, socioeconomic status. A Wald z-statistic was used for p-values.

There may be some missing data while not all participants reported complete information on health questionnaires
